# Adiponectin, Omentin, Ghrelin, and Visfatin Levels in Obese Patients with Severe Obstructive Sleep Apnea

**DOI:** 10.1155/2018/3410135

**Published:** 2018-07-29

**Authors:** Dong-mei Zhang, Xing-long Pang, Rong Huang, Feng-ying Gong, Xu Zhong, Yi Xiao

**Affiliations:** ^1^Department of Respiratory Medicine, Peking Union Medical College Hospital, Chinese Academy of Medical Sciences & Peking Union Medical College, No. 1 Shuaifuyuan Street, Dongcheng District, Beijing 100730, China; ^2^Department of Endocrinology, Key Laboratory of Endocrinology of Ministry of Health, The Translational Medicine Center, Peking Union Medical College Hospital, Chinese Academy of Medical Sciences & Peking Union Medical College, No. 1 Shuaifuyuan Street, Dongcheng District, Beijing 100730, China

## Abstract

**Objectives:**

Obstructive sleep apnea (OSA) is closely associated with obesity, insulin resistance, and inflammation. Adiponectin, omentin, ghrelin, and visfatin are adipokines involved in insulin sensitivity or regulation of inflammatory disease. This study aims to clarify the relationship between OSA and associated adipokines.

**Patients and Methods:**

Thirty overweight male patients with severe OSA and twenty controls underwent standard diagnostic polysomnography (PSG), and 10 patients underwent overnight continuous positive airway pressure (CPAP) treatment. Blood samples were collected in the morning after PSG or CPAP procedures.

**Results:**

Among the investigated adipokines, only plasma omentin levels of patients with OSA were significantly lower than those of control subjects (442.94 ± 191.89 ng/ml versus 573.52±228.67 ng/ml, p=0.034) and levels did not change after CPAP treatment. In patients with OSA, omentin levels were positively correlated with high-density lipoprotein cholesterol (HDL) levels (r=0.378, p=0.007), adiponectin levels (r=0.709, p<0.001), percentage of sleep at the rapid eye movement (REM) stage (r=0.307, p=0.003), and average and minimum SpO_2_ (p=0.041, 0.046, respectively) and negatively with hypersensitive C-reactive protein (hsCRP, r=-0.379, p=0.007) and apnea-hypopnea index (AHI, r=-0.315, p=0.026). However, plasma concentrations of adiponectin, ghrelin, and visfatin in patients with OSA did not significantly differ from those of the control or correlate with sleep parameters and CPAP treatment.

**Conclusions:**

Patients with OSA have decreased omentin levels, which are associated with sleep parameters, including AHI, SpO_2_, percentage of REM sleep, hsCRP, HDL, and adiponectin levels.

## 1. Introduction

Obstructive sleep apnea syndrome (OSA) is characterized by recurrent episodes of upper airway obstruction, which leads to increased negative intrathoracic pressure, sleep fragmentation, and intermittent hypoxia during sleep. Considerable evidences suggest that OSA magnifies metabolic, inflammatory, and cardiovascular dysfunctions and may have implications in hypertension, atrial fibrillation, diabetes, and pulmonary hypertension. Currently, about 14% adult males and 5% females have an AHI ≥ 5 with symptoms of daytime sleepiness [1].

OSA imposes a large disease burden and requires timely diagnosis and treatment. Obesity is the most significant predisposing factor for OSA, and an increase of 6 kg/m^2^ in body mass index (BMI) correlates with a fourfold risk of developing OSA [2]. Hormones produced by adipose tissue may be involved in the mechanism of OSA. Leptin, the most widely studied adipocyte-derived hormone, plays a key role in body-weight regulation and central respiratory control [3]. Patients with obesity have higher levels of leptin and display leptin resistance.

Recently, other adipocyte-derived hormones, such as adiponectin, omentin, and visfatin, were consecutively discovered and reported to play roles in energy metabolism and the regulation of inflammation. OSA may contribute to decreased serum adiponectin levels, [4, 5] and patients with OSA display elevated circulating omentin levels [6]. In patients with severe OSA, visfatin levels have been suggested to correlate with characteristics of sleep architecture [7]. In analogy with leptin, ghrelin stimulates hunger and food intake, promotes fat accumulation, [8] and is related to obesity in patients with OSA [9]. Few studies have investigated the direct association between adipocyte hormones and OSA, and literatures have presented conflicting theories. Here, we compare levels of plasma adiponectin, omentin, ghrelin, and visfatin in patients with OSA (as well as control participants) and clarify the relationships between adipokines and sleep disorders.

## 2. Patients and Methods

### 2.1. Patients

Fifty adult males (18-70 years old), including 30 patients with severe OSA patients and 20 healthy controls, were consecutively recruited from the Sleep Disorders Center of Peking Union Medical College Hospital (Beijing, China). The duration of this study was about 6 months. The newly diagnosed patients were overweight (BMI>24 kg/m^2^) or obese (BMI>30 kg/m^2^). OSA was defined as apnea-hypopnea index (AHI) more than 5 times per hour and with symptoms of daytime sleepiness. Those who displayed AHI greater than 30/h were diagnosed with severe OSA, and those with lower scores were classified as mild/moderate OSA.

Subjects were excluded from our study for the following reasons: the presence of other sleep disorders (e.g., central sleep apnea, upper airway resistance, or narcolepsy), current treatment with continuous positive airway pressure (CPAP), presence of craniofacial and anatomical abnormalities, history of upper and/or lower airway surgery, significant neuromuscular disease, renal disease, endocrine disease, heart failure, any acute infectious processes, inflammatory or autoimmune diseases, or corticosteroid intake.

All subjects completed a detailed clinical questionnaire and Epworth sleepiness scale (ESS). The following data were collected: body weight, height, neck circumference, waist and hip circumference, and blood pressure before the sleep study. In addition, participants completed an informed written consent that was approved by the Ethics Committee of the Institutional Review Board in Peking Union Medical College Hospital (protocol number ZS-1107).

### 2.2. Polysomnographic Evaluation and CPAP Therapy

Full-night diagnostic polysomnography (PSG, Rembrandt, USA) was administrated to participants from 22:00 to 6:00 (the second day). The following parameters were recorded (Rembrandt Manager 7.5 Master software, Medicare, USA): electroencephalogram (EEG), electrooculogram (EOG), submental electromyogram (EMG), electrocardiogram, chest and abdominal wall motion, airflow by nasal pressure, and finger pulse oximetry. Sleep architecture, arousals, and respiratory events were analyzed according to the standard criteria recommended by American Academy of Sleep Medicine [10]. Apnea was defined as a drop of airflow ≥90% for at least 10 seconds, and hypopnea was defined as a decrease in airflow ≥30% for at least 10 seconds accompanied by oxygen desaturation or arousal. AHI was calculated by the total number of apnea and hypopnea episodes divided by total sleep time. Nocturnal oxygen desaturation was evaluated by minimum SpO_2_, average SpO_2_, and sleep time during which SpO_2_ <90%. All data were scored using a double-blind protocol.

Patients with OSA under CPAP treatment received a second PSG with auto-CPAP titration (Autoset® CS, ResMed, Mönchengladbach, Germany). Positive titration was started with the pressure set at 4 cm H_2_O. The therapy was considered effective for subjects who displayed an AHI less than 10 events per hour or 75% decrease from baseline.

### 2.3. Laboratory Assessment

Venous blood was collected between 6 a.m. and 7 a.m. following PSG or CPAP therapy. Samples were collected into pyrogen-free tubes (Vacutainer, BD Diagnostics, NJ, USA) and centrifuged (3000 g, 15 min) at 4°C, and the supernatant plasma was stored at -80°C until use. All assessments were completed in the Biochemistry Laboratory and Key Laboratory of Endocrinology in PUMCH. Fasting blood glucose (FBG), plasma triglycerides (TG), total cholesterol (TC), high-density lipoprotein cholesterol (HDL-C), low-density lipoprotein cholesterol (LDL-C), and hypersensitive C-reactive protein (hsCRP) were determined using an automated biochemistry instrument (Hitachi 7060, Tokyo, Japan). Plasma adiponectin, omentin, ghrelin, and visfatin levels were measured using a sandwich enzyme-linked immunosorbent assay (ELISA). All measurements were performed at the same time for three replicates.

### 2.4. Statistical Analysis

Data were analyzed using SPSS software version 21.0 (SPSS, Chicago, IL, USA) and GraphPad Prism 6.02 (GraphPad Software Inc, La Jolla, USA). Results were expressed as mean ± standard deviation. Unpaired *t*-tests were used to compare mean values between OSA and control groups. Subsequently, Pearson's correlation coefficient analyses were conducted to quantify associations between adipokine levels and clinical/biochemical information. Paired *t*-tests were used to compare data pre- and post-CPAP treatment.* P*<0.05 was considered statistically significant.

## 3. Results

### 3.1. Baseline Information

Basal characteristics of the 50 subjects are shown in Table 1. No significant differences were observed in mean age and BMI between OSA and control group. Compared with controls, neck circumference and waist/hip radio were significantly higher in patients with OSA (p=0.017, p=0.002, respectively). No significant differences were discovered in metabolic indices between groups, which included fasting plasma glucose, TG, TC, HDL, and LDL. hsCRP, a parameter of inflammation, showed a significant increase in patients with OSA (p=0.036).

The OSA group exhibited severe degrees of AHI (61.48±15.00/h) and significantly lower nocturnal oxygen desaturation in comparison with the control group. Furthermore, patients with OSA displayed poorer sleep quality, which was observed as longer stage N2 and shorter stage N3 (*p*<0.001), even though the NREM and REM sleep of patients with OSA were similar to those of controls. Moreover, patients with OSA suffered from obvious daytime sleepiness, as assessed by ESS (*p*=0.032).

### 3.2. OSA Patients Have a Decreased Omentin Levels

**Figure 1:**
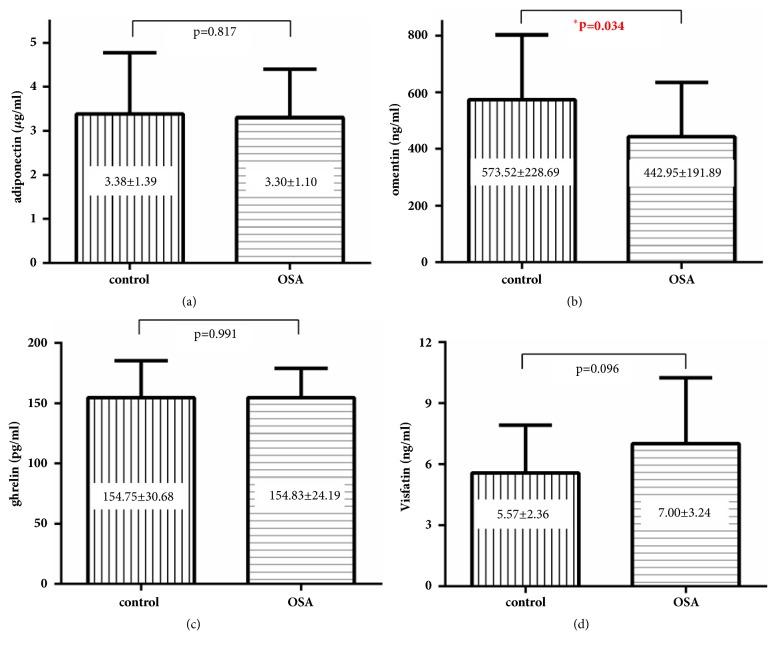
Concentration of adiponectin, omentin, ghrelin, and visfatin in study subjects.

**Figure 2:**
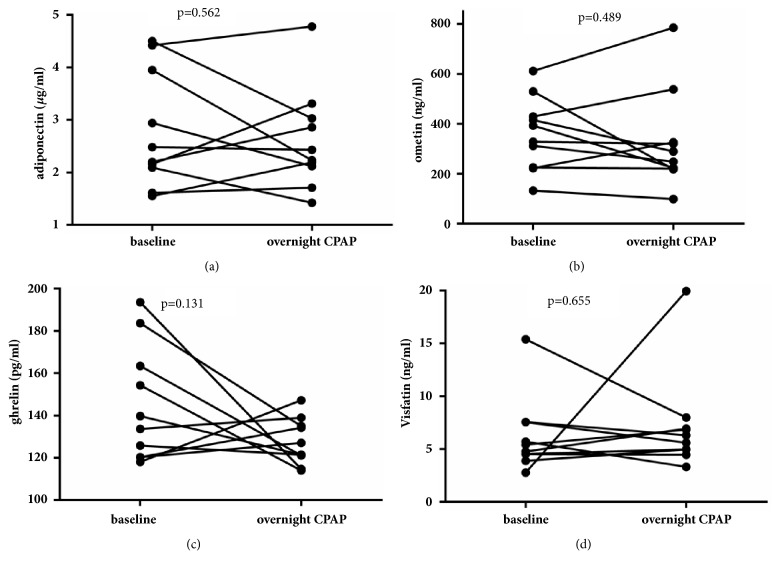
Concentration of adiponectin, omentin, ghrelin, and visfatin after overnight CPAP therapy.

As shown in Figure 1, only omentin levels were significantly decreased in patients with severe OSA (442.94±191.89 ng/ml versus 573.52±228.67 ng/ml,* p*=0.034). No significant differences were observed in adiponectin, ghrelin, and visfatin levels between patients with OSA and the control group. After overnight CPAP treatment, even though AHI substantially decreased (59.63±23.00 versus 7.50±4.76,* p*<0.001) and oxygen saturation improved (mean SpO_2_ 90.33±6.24 versus 95.90±1.33,* p*=0.005, min SpO_2_ 70.83±14.97 versus 89.25±3.89,* p*<0.001), levels of adipokines other than omentin did not significantly change (p=0.278, 0.489, 0.106, 0.407, 0.184 respectively, Figure 2).

### 3.3. Correlations between Omentin Levels and Sleep Characteristics

**Figure 3:**
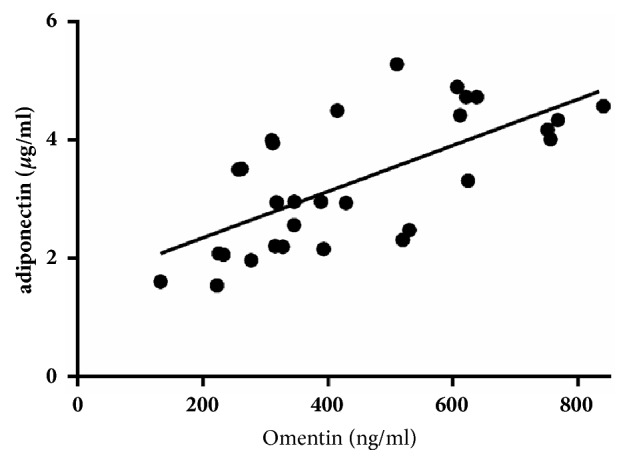
Correlations between omentin and adiponectin.

In all subjects, omentin levels significantly correlated with HDL levels (r=0.415, p=0.023), while no associations were observed between omentin and anthropometric variables (age, BMI, neck circumference, waist-hip ratio, and mean blood pressure) and other metabolic data (FPG, TC, TG, and LDL). Although we found differences in neck circumference and Waist-Hip ratio between groups, only a relationship between neck circumference and visfatin was revealed. With respect to inflammation factors, there was a significant negative association between omentin levels and hsCRP (r=-0.379, p=0.007). Furthermore, a strong positive correction was found between omentin and adiponectin levels (r=0.709, p<0.001, Figure 3). Among correlations between omentin levels and sleep characteristics, the percentage of REM stage (r=0.307, p=0.030) and minimum and average SpO_2_ significantly correlated with omentin levels (p=0.046, p=0.041, respectively). AHI was negatively correlated with omentin (r=-0.315, p=0.026), and no associations were observed between omentin and time spent in SpO_2_<90% or percentages of other sleep stages (Table 2).

## 4. Discussion

The major finding of our study was that plasma omentin levels were significantly lower in patients with OSA, compared to controls, and that neither adiponectin, ghrelin, nor visfatin differs between OSA and control group.

Accumulating evidences have shown that omentin is a novel link between inflammation, diabetes, obesity, and cardiovascular disease, and probing its role may benefit patients with metabolic syndromes [11, 12]. Several studies have reported inconsistent omentin results, including either elevated omentin levels in patients with OSA [6, 13] or decreased levels [14, 15]. Based on some studies, which demonstrated that decreased omentin levels are associated with increased obesity, insulin resistance, and comorbidities [12, 16–18], we hypothesize that patients with OSA have lower omentin levels. Furthermore, the effects of CPAP treatment on omentin levels were consistent between two published investigations, which reported that omentin returns to healthy levels three months after therapy [13, 15]. In our study, one night of CPAP treatment failed to restore the omentin level in patients with OSA.

We found that plasma omentin levels most significantly correlated with adiponectin levels, and after adjusting for adiponectin, the correction between omentin levels and HDL was abolished. A large population-based cross-sectional study enrolling 1092 participants also suggested that adiponectin may mediate omentin and HDL cholesterol levels [19]. A significant positive correlation between these variables was observed in diet-induced obese rats [20], and our findings support the hypothesis that adiponectin upregulates omentin and then influences lipid metabolism, although the precise details require further elucidation.

On the other hand, plasma omentin levels were negatively correlated with hsCRP and AHI and positively associated with REM duration. This finding suggests that the anti-inflammation function of omentin is independent from adiponectin, and sleep apnea may influence the secretion of omentin, which could be involved in OSA pathophysiology. In a previous study, Uygur F et al. also observed a negative correlation between circulating omentin levels and AHI, and a positive correlation between CRP and mean SaO_2_ [15]. CRP and hsCRP are the most widely used inflammation biomarkers in patients with OSA, and increased hsCRP levels may be due to a loss of anti-inflammation function when omentin expression decreases. Based on our data, some adipokines, such as omentin, could provide a reference in OSA diagnosis, as a biomarker, as well as monitoring the development of OSA and preventing future comorbidities.

Given that OSA is strongly associated with hypertension, ischemic heart disease, coronary artery disease, and stroke, this disorder is regarded as an independent risk factor for cardiovascular diseases (CVDs) [21]. The pathogenesis of CVDs is multifactorial and includes the activation of inflammatory pathways, endothelial dysfunction, and metabolic dysregulation, and, here, omentin was observed to have a protective function in CVDs. Previously, it has been reported that Omentin-1 is negatively associated with endothelial dysfunction, arterial stiffness, and coronary artery disease [22–24]. Furthermore, omentin maintains endothelial cell function and promotes revascularization, in response to ischemia, through the Akt-eNOS signaling pathway [25]. We suppose that lower omentin levels may help identify patients with OSA and help explain why OSA patients have an increased risk of CVDs.

Furthermore, OSA is known to induce severe insulin resistance, and a vast body of literature has reported the relationship between OSA and T2DM [26–30]. Additionally, OSA severity is associated with increased insulin resistance [26] and poor glucose control [30], and concentrations of omentin are reduced in patients with obesity, polycystic ovary syndrome, and type 2 diabetes, all of which are associated with insulin resistance [16, 17, 31–33]. Omentin is deemed to play a role in insulin resistance by enhancing subcutaneous glucose transport [34], and available research indicates a positive correlation between plasma adiponectin and omentin in regulating insulin sensitivity and glucose and lipid profiles [19, 20]. In our study, all subjects displayed normal FPG, and no differences in FPG and adiponectin levels were observed. However, we observed significant positive correlation between adiponectin and omentin levels, which may be due to a floor effect of glucose levels.

Adiponectin is a messenger that connects adipose tissue with other organs and believed to be involved in especially insulin resistance, for example, type 2 diabetes. In Al Mutairi's study [35], lower adiponectin was found to associate with OSA severity, and the FBG and homeostasis model assessment of insulin resistance (HOMA-IR) also increase with OSA severity. However, majority of our subjects had normal FBG levels, and only three individuals displayed FBG levels from 7.00 to 7.20 mmol/L. While no correlation was observed between FBG and adiponectin levels, it may be premature to conclude that adiponectin could function as a biomarker for OSA with insulin resistance.

Ghrelin was first discovered from rat stomach extracts [36] and plays key roles in stimulating appetite, promoting food intake, and improving fat accumulation. Ghrelin levels are commonly reduced in obese individuals and scale inversely with BMI [8]. While the relationship between plasma ghrelin and OSA remains controversial. Harsch et al. [37] found that basal plasma ghrelin levels are significantly elevated in patients with OSA and rapidly decreased after 2 nights of CPAP treatment. Alternatively, Ulukavak et al. [38] discovered that plasma ghrelin was not associated with OSA but with BMI. Furthermore, Liu [9] reported that ghrelin levels were significantly lower in patients with OSA than those only with normal weight and overweight one; and they revealed that ghrelin negatively correlates with BMI but not with sleep parameters. In our study, no differences in ghrelin levels were observed between patients with OSA and overweight control patients. After overnight CPAP treatment, ghrelin expression did not significantly change, which suggests OSA may not necessarily play a role in ghrelin regulation.

Visfatin, first reported in 2005, is an insulin-mimicking adipokine. It is considered a mediator between obesity and insulin resistance [39]. Plasma visfatin concentration increases with obesity progression, and visfatin may contribute to the development of metabolic syndromes. So far, only three studies have published visfatin data from patients with OSA and conflicting conclusions exist. Trakada et al. [7] found no differences in visfatin expression between OSA patients and healthy controls, while Kiskac et al. [40] reported visfatin decrease in patients with severe OSA. Both Trakada [7] and Hayes [41] identified an association between short sleep time and visfatin upregulation in patients with severe OSA. However, in our study, no significant differences in visfatin were found between groups. Therefore, the correlation between visfatin and lipid profile remains elusive, and more research is required to determine the potential of visfatin as a surrogate marker of metabolic syndromes in patients with OSA.

There have been several randomized sham-controlled trials of CPAP that have also investigated adipokine changes. Ng SS et al. [42] found that adiponectin and irisin levels change significantly following 3 months of therapeutic CPAP versus subtherapeutic CPAP therapy, which is consistent with our observations. However, Li AM et al. [43] performed a survey on children with habitual snoring and symptoms suggestive of OSA and concluded that subjects with OSA did not have significantly different adiponectin and leptin concentrations from those without OSA, across both obese and nonobese individuals. Furthermore, systolic BP, age, high-density lipoprotein cholesterol, and BMI z-score independently associate with adiponectin, whereas diastolic BP, triglyceride, height, and BMI z-score independently associate with leptin concentration. This inconsistency with our study may be due to different sample ages. Eun YG et al. [44] reported that, after uvulopalatopharyngoplasty and radiofrequency tongue base reduction, OSA patients had decreased leptin and increased adiponectin expression, which suggests adipokines may respond to a single surgery within a range of OSA severities (mild, moderate, and severe) better than in patients with severe OSA.

Our study has a few limitations. First, the sample size was small, and since only patients with severe OSA were included, our investigation lacks patients with mild to moderate OSA. Moreover, we did not examine the relationship between insulin level and HOMA-IR. Finally, we only compared the acute (overnight) effect of CPAP treatment and long-term monitoring of the effects of CPAP therapy which merit further investigation.

## 5. Conclusions

Plasma omentin levels were lower in patients with severe OSA, compared to matched controls. Omentin levels are associated with sleep parameters, including AHI, SpO_2_ and percentage of REM in total sleep time, hsCRP, HDL, and adiponectin levels. Our findings suggest that, in overweight and obese individuals, omentin may be a predictive and diagnostic factor of sleep disorders, inflammation, and lipid metabolic disorders.

## Figures and Tables

**Table 1:** Baseline clinical, biochemical, and polysomnographic characteristics of the study subjects.

	**Control n=20**	**OSA n=30**	**p**
Age	36.10±13.67	40.73±8.90	0.192
BMI kg/m^2^	27.55±2.97	28.85±2.62	0.108
NC cm	41.55±2.14	43.03±2.05	0.017
WHR	0.92±0.043	0.97±0.06	0.002
MBP	95.72±9.17	100.28±10.59	0.122
FPG mmol/L	5.12±0.73	5.44±0.93	0.202
TC mmol/L	4.80±0.77	5.05±0.93	0.315
TG mmol/L	1.97±2.14	3.31±3.89	0.169
HDL mmol/L	1.08±0.19	1.04±0.19	0.477
LDL mmol/L	3.03±0.74	3.06±0.76	0.909
hsCRP mg/L	1.19±1.14	2.09±1.80	0.036
AHI	1.93±1.38	61.48±15.00	<0.001
Mean SpO_2_,%	95.55±1.24	91.40±4.55	<0.001
Min SpO_2_, %	89.85±3.44	71.77±11.45	<0.001
SpO_2_<90%, %	0.09±0.14	23.46±20.17	<0.001
REM, %	15.47±8.06	13.55±6.06	0.343
NREM, %	76.31±8.51	79.18±11.13	0.333
N1, %	9.18±7.06	7.30±5.71	0.307
N2, %	54.12±7.84	66.32±12.18	<0.001
N3, %	13.02±7.65	5.57±5.69	<0.001
ESS	7.90±4.48	11.10±5.35	0.032

BMI: body mass index, NC: neck circumference, WHR: waist-hip ratio, MBP: mean blood pressure, FPG: fasting plasma glucose, TC: total Cholesterol, TG: triglycerides, HDL: high-density lipoprotein cholesterol, LDL: low-density lipoprotein cholesterol, hsCRP: hypersensitive respective Protein. AHI: apnea-hypopnea index, Mean SpO_2_: average SpO_2_, Min SpO_2_: minimum SpO_2_, SpO_2_<90%: percentage of total sleep time spent with SpO_2_<90%, REM: percentage of rapid eye movement sleep in total sleep time, NREM: percentage of rapid eye movement sleep in total sleep time, N1, N2, N3: percentage of stage N1, stage N2, and stage N3 in total sleep time, and ESS: Epworth sleepiness scale.

**Table 2:** Correlations between adipokines levels and characteristics.

	**Omentin**		**Adiponectin**		**Ghrelin**		**Visfatin**	
	**r**	**p**	**r**	**p**	**r**	**p**	**r**	**p**
**Age**	.111	.441	-.028	.845	-.154	.284	.011	.941
**BMI (kg/m2)**	-.222	.121	.064	.659	.143	.321	.077	.594
**NC (cm)**	-.135	.351	.015	.919	.147	.308	.349	.013^*∗*^
**WC (cm)**	-.228	.111	-.101	.486	.031	.831	.121	.402
**WHR**	-.197	.171	-.159	.270	.073	.617	.063	.665
**FPG (mmol/L)**	-.180	.211	-.186	.195	-.075	.604	.023	.874
**TC (mmol/L)**	.029	.843	.023	.875	-.022	.880	.211	.142
**TG (mmol/L)**	-.141	.328	-.225	.115	-.105	.468	.317^*∗*^	.025^*∗*^
**HDL (mmol/L)**	.378^*∗∗*^	.007^*∗∗*^	.286^*∗*^	.044^*∗*^	.123	.394	-.082	.573
**LDL (mmol/L)**	.099	.495	.225	.116	.098	.500	-.124	.391
**hsCRP (mg/L)**	-.379^*∗∗*^	.007^*∗∗*^	-.209	.145	.216	.132	.002	.990
**NREM**%	-.054	.708	-.119	.409	-.084	.562	.048	.741
**REM**%	.307^*∗*^	.030^*∗*^	.143	.321	.126	.382	-.109	.451
**Mean SpO2**%	.290^*∗*^	.041^*∗*^	.237	.098	-.104	.471	-.126	.382
**Min SpO2**%	.284^*∗*^	.046^*∗*^	.164	.255	-.081	.575	-.201	.161
**AHI**	-.315^*∗*^	.026^*∗*^	-.120	.407	-.021	.885	.295^*∗*^	.038^*∗*^
**ESS**	-.168	.244	-.046	.750	.176	.221	.119	.409
**N1**	-.103	.477	-.044	.761	-.031	.831	.035	.808
**N2**	-.050	.731	-.108	.457	-.174	.226	.128	.374

## Data Availability

The data used to support the findings of this study are available from the corresponding author upon request.
